# Predictors of upper trapezius pain with myofascial trigger points in food service workers: The STROBE study

**DOI:** 10.1097/MD.0000000000007252

**Published:** 2017-06-30

**Authors:** Ui-Jae Hwang, Oh-Yun Kwon, Chung-Hwi Yi, Hye-Seon Jeon, Jong-Hyuck Weon, Sung-Min Ha

**Affiliations:** a Department of Physical Therapy, Graduate School, Yonsei University; b Department of Physical Therapy, College of Health Science, Laboratory of Kinetic Ergocise Based on Movement Analysis, Yonsei University, Wonju; c Department of Physical Therapy, Joongbu University, Chubu-myeon, Geumsan-gun, Chungcheongnam-do; d Department of Physical Therapy, College of Health Science, Sangji University, Wonju, South Korea.

**Keywords:** food service worker, influencing factor, multiple regression, myofascial trigger points, upper trapezius pain

## Abstract

Shoulder pain occurs commonly in food service workers (FSWs) who repetitively perform motions of the upper limbs. Myofascial trigger points (MTrPs) on the upper trapezius (UT) are among the most common musculoskeletal shoulder pain syndromes. This study determined the psychological, posture, mobility, and strength factors associated with pain severity in FSWs with UT pain due to MTrPs.

In this cross-sectional study, we measured 17 variables in 163 FSWs with UT pain due to MTrPs: a visual analog scale (VAS) pain score, age, sex, Borg rating of perceived exertion (BRPE) scale, beck depression inventory, forward head posture angle, rounded shoulder angle (RSA), shoulder slope angle, scapular downward rotation ratio, cervical lateral-bending side difference angle, cervical rotation side difference angle, glenohumeral internal rotation angle, shoulder horizontal adduction angle, serratus anterior (SA) strength, lower trapezius (LT) strength, bicep strength, and glenohumeral external rotator strength, in 163 FSWs with UT pain due to MTrPs.

The model for factors influencing UT pain with MTrPs included SA strength, age, BRPE, LT strength, and RSA as predictor variables that accounted for 68.7% of the variance in VAS (*P* < .001) in multiple regression models with a stepwise selection procedure. The following were independent variables influencing the VAS in the order of standardized coefficients: SA strength (β = −0.380), age (β = 0.287), BRPE (β = 0.239), LT strength (β = −0.195), and RSA (β = 0.125).

SA strength, age, BRPE, LT strength, and RSA variables should be considered when evaluating and intervening in UT pain with MTrPs in FSWs.

## 1 Introduction

Cooks and restaurant workers are at high risk for work-related musculoskeletal disorders (WMSDs) because of the high strain on the body associated with preparing raw materials and cooking.^[1–3]^ A high prevalence of WMSDs has been reported among food service workers (FSWs) at Chinese restaurants in both Taiwan and Hong Kong. A survey that focused on the high prevalence of WMSDs among FSWs confirmed that, of 905 participants, the shoulders (57.9%), neck (54.3%), and lower back/waist (52.7%) were more affected than other body sites (22.3–46.5%).^[4,5]^

Myofascial trigger points (MTrPs) are one of the most common musculoskeletal pain conditions.^[6]^ MTrPs are hyperirritable nodules of tenderness in a palpable taut band of a skeletal muscle,^[7–12]^ and they make a major contribution to the generation of pain and motor dysfunction.^[13–15]^ In the upper quadrant, postural muscles, in general, and the upper trapezius (UT), in particular, are most affected by MTrPs.^[16–18]^

To determine a specific treatment approach for shoulder pain, it is important to perform an evaluation based on an examination of neck and shoulder posture, mobility or range of motion (ROM), strength of the rotator cuff muscle, and strength of the scapular rotator.^[19]^ The combination of passive testing to determine the length of the tissue and muscle testing to determine strength helps to identify muscle imbalances. Many causes of UT pain have been suggested. Regarding the neck and shoulder posture, a forward head posture (FHP)^[20]^ and abnormal scapula alignment^[21–23]^ can be a source of shoulder pain, as confirmed in studies of the factors influencing UT pain with MTrPs.^[24]^Joint alignment is an indicator of FHP shortening of the UT and levator scapulae muscles, which results in an elevated scapula, and abnormal alignments need to be corrected to allow for optimal motion.^[19]^ MTrPs are hyperirritable spots of a skeletal muscle associated with a hypersensitive palpable nodule in a taut band that produces specific patterns of referred pain associated with a restricted ROM.^[8,9,25]^ The UT acts as an extrinsic cervical rotator, extensor, and lateral flexor. The UT can affect the motion of the cervical spine through its attachment to the ligamentum nuchae and spinous processes of the vertebrae.^[26,27]^ Posterior shoulder tightness is seen in glenohumeral internal rotation (GIR) deficits and limited shoulder horizontal adduction.^[28–30]^ With limited glenohumeral joint motion, scapulothoracic joint movement is more dominant than glenohumeral joint movement.^[23]^ Regarding the strength of the scapular rotators, imbalance of the scapular upward rotators relative to overload on the UT influences UT pain with MTrPs.^[31–33]^ Deficient control by the serratus anterior (SA), causing impairment in the timing and range of scapular motion, can cause stress at the glenohumeral joint. The main muscles thought to facilitate scapular upward rotation and posterior tilt are the lower trapezius (LT) muscle and SA.^[34,35]^ It has been suggested that muscle imbalances in the scapulothoracic region occur when the UT becomes tight and the SA and the LT become weak.^[36,37]^ Regarding the strength of the external glenohumeral rotators, weakness of the external rotators of the humerus related to overload of the UT influences UT pain with MTrPs.^[38,39]^ Motions that occur during shoulder elevation include excessive anterior or superior translation of the humeral head on the glenoid fossa, noncorrective glenohumeral external rotation, and decreases in normal scapular upward rotation and posterior tipping on the thorax.^[40]^ Furthermore, several factors have been proposed to influence MTrPs, psychological and mechanical factors.^[19]^ Psychosocial risk factors include monotonous or boring work tasks, high time pressure, low social support, and low job satisfaction with performance of work tasks.^[41,42]^ The focus on pain modulation has occurred simultaneously with increasing studies investigating the impact of psychological factors on the control of pain.^[43,44]^

In previous studies, ergonomic surveys (e.g., the Rapid Upper-Limb Assessment and Ovako Working Posture Analysis System) or questionnaire surveys on fatigue and discomfort of FSWs have been conducted to investigate the risk of WMSDs.^[1–3,5,45]^ Although such surveys have been performed for FSWs, no study has determined the factors (e.g., incorrect posture, limited mobility, lack of strength, and psychological factors) that influence UT pain with MTrPs in FSWs.

Therefore, this study determined the extent to which psychological, posture, mobility, and strength factors are associated with pain severity in FSWs with UT pain for MTrPs.

## 2 Methods

### 2.1 Subjects

**Figure 1:**
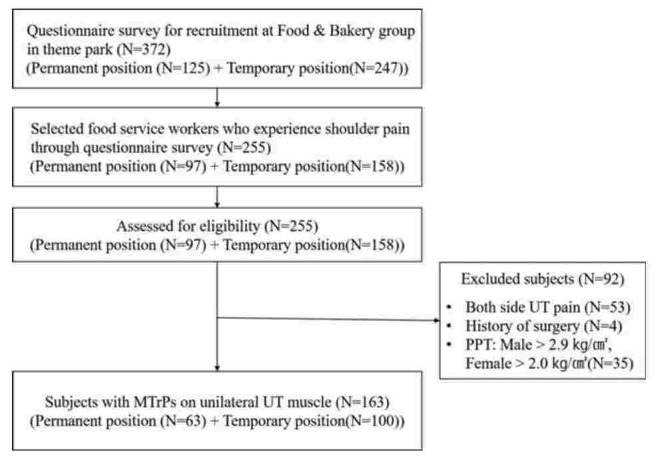
Flow diagram of study participant selection.

Participants were recruited through a questionnaire to confirm their experience of UT pain as FSWs in a theme park. In total, 163 subjects with UT pain with MTrPs participated, among 372 workers in a food and beverage group in the theme park. A flowchart for recruitment of the subjects in the present study is provided in Figure 1. To be included in this study, participants must have had all of the following: duration of work in food service longer than 6 months, unilateral nontraumatic shoulder pain, experience of shoulder pain for more than 2 months, experience of tenderness of UT more than twice over the past week, latent MTrPs in the UT muscle through measurement of the pressure-pain threshold (PPT) for males of <2.9 kg/cm^2^ and for females of <2.0 kg/cm^2^,^[46]^ and a visual analog scale (VAS) score over 30 mm. Exclusion criteria were a prior diagnosis of shoulder instability, shoulder fractures, any systemic disease, a history of surgery in the shoulder, and examination suggesting the presence of neurological diseases, internal diseases, or psychiatric disorders.^[10]^ Participant characteristics are shown in Table 1.

**Table 1:**
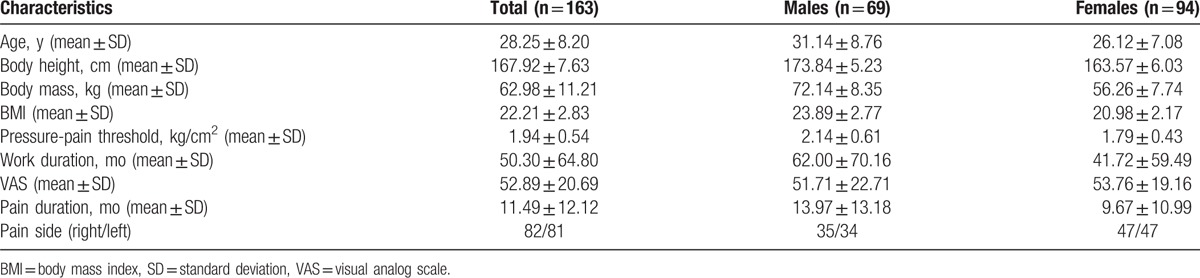
Subject characteristics.

The study protocol and informed consent document were approved by the Yonsei University Wonju Institutional Review Board. Prior to testing, the investigator explained the entire procedure, and all subjects voluntarily gave their informed consent.

### 2.2 Outcome measures

#### 2.2.1 Visual analog scale

A VAS is a valid and reliable measurement tool for evaluation of pain intensity in clinical research and at clinical stations.^[47,48]^ It consists of a 100-mm horizontal line anchored by 2 verbal descriptors.^[47,48]^ The anchor at one end is “no pain (score 0),” and that at the other is “worst pain imaginable (score 100).”^[47,48]^ The subject is asked to mark a single spot on the horizontal line indicating his/her current level of UT pain.^[47,48]^

#### 2.2.2 Borg rating of perceived exertion scale

The well-known RPE scale, from 6 to 20, was used.^[49–51]^ Subjects were instructed to check their exertion of work intensity and rate their perception of themselves on a scale between 6 and 20. The examiner explained that the subjects could check the matching exertion score and verbal level. In ergonomic investigations of work tasks, perceived exertion is used in studies of heavy aerobic work.^[52]^

#### 2.2.3 Beck depression inventory

The beck depression inventory (BDI) is a well-known and widely used depression scale.^[53–55]^ The BDI consists of 21 items based on attitudes and symptoms that Beck observed to be common among depressed patients and uncommon among the nondepressed. The statements are ranked to reflect the range of severity of the symptom from neutral to maximal severity.

#### 2.2.4 Posture analysis

##### 2.2.4.1 Forward head posture angle

**Figure 2:**
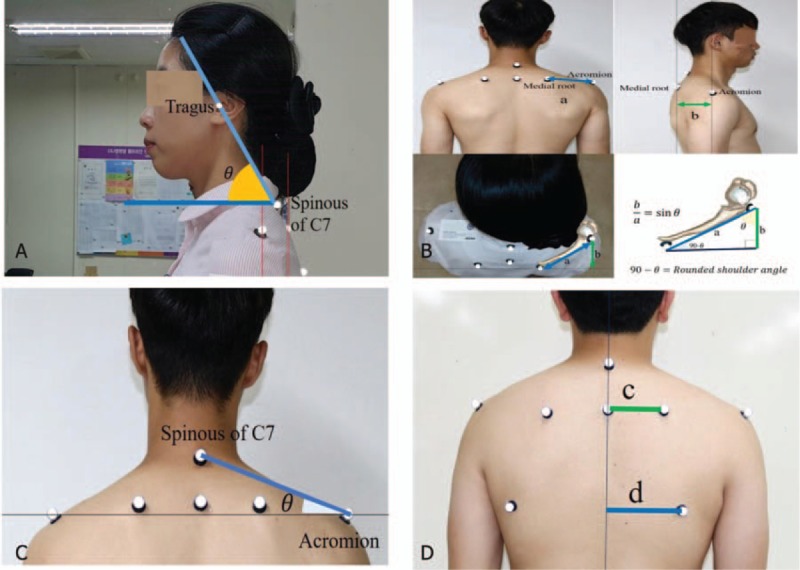
Posture analysis: (A) measurement of the forward head posture angle, (B) calculation of the rounded shoulder angle (a: distance between the root of the scapula and the acromion, b: the distance between the acromion and the horizontal line in the root of the scapula), (C) measurement of the shoulder slope angle in posterior view, (D) calculation of the scapular downward rotation ratio (c: distance between mid-line and root of scapula, d: distance between mid-line and inferior angle).

FHP was assessed using a digitized, lateral-view photograph of the subject in his/her usual standing posture (Fig. 2).^[56]^ The tragus of the subject's ear was marked, and a reflexive marker was attached to the skin overlying the C7 vertebra. Once the photograph was obtained, we used ImageJ software (National Institutes of Health, Bethesda, Maryland) to measure FHP, quantified by the craniovertebral angle (the angle between the horizontal line passing through C7 and a line extending from the tragus of the ear to C7).^[57,58]^

##### 2.2.4.2 Rounded shoulder angle

Rounded shoulder angle (RSA) was assessed using a digitized, transverse-view photograph of the subject in his/her usual standing posture (Fig. 2). Calculation of RSA requires 2 distances. One distance, in the transverse plane, from a horizontal line in medial roots of the scapula to the acromion was measured based on the reference of a business card (size: 9 × 4 cm), in a transverse-view photograph. The other distance, in the transverse plane, from the root of the scapula to the acromion was measured based on the reference of a business card, in a transverse-view photograph. The 2 distances were calculated using ImageJ software (National Institutes of Health). A triangle was made from the 2 lines, and the angle can be calculated with a sine function. For example, distance B (the height of a right-angled triangle)/distance A (the hypotenuse of a right-angled triangle) gives sin θ (Fig. 2). θ, composed of the 2 distances, is one apex of a right-angled triangle. Then 90 − θ is the other apex of the right-angled triangle. We defined RSA as 90 − θ.

##### 2.2.4.3 Shoulder slope angle

Shoulder slope angle (SSA) was measured using a digitized, posterior-view photograph of the subject in his/her usual standing posture (Fig. 2). For SSA measurements, the examiner palpated the subject's scapula and attached a reflexive marker on 2 landmarks: the spinous process of the 7th cervical vertebrae and the acromion. In the photograph, we drew a horizontal line with the acromion and a line between the spinous process of the 7th cervical vertebrae and acromion. We defined the SSA as the angle between the 2 lines. SSA was calculated with ImageJ software (National Institutes of Health).

##### 2.2.4.4 Scapular downward rotation ratio

The scapular downward rotation ratio (SDRR) was calculated using a digitized, posterior-view photograph of the subject in his/her usual standing posture (Fig. 2). For this measurement, the examiner palpated the subject's scapula and attached reflexive markers on 4 landmarks: the spinous process of the 7th cervical vertebrae, the spinous process of the 2nd thoracic vertebrae, the medial root of the scapular spine, and the inferior angle. A vertical line was drawn between the spinous process of the 7th cervical vertebrae and the spinous process of the 2nd thoracic vertebrae. In addition, 2 horizontal distances were measured between the vertical line and the root of scapula and inferior angle using ImageJ software (National Institutes of Health; Fig. 2). We defined SDRR as the distance between the root of the scapula and a vertical line/the distance between the inferior angle and vertical line.

#### 2.2.5 Measurements of range of motion

##### 2.2.5.1 Cervical lateral-bending and rotation side difference angle

**Figure 3:**
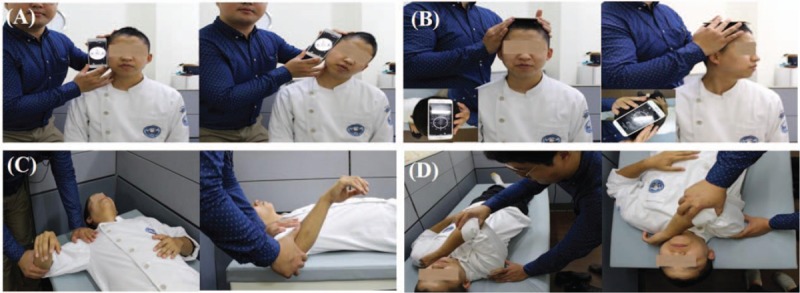
Measurement of the range of motion: (A) cervical lateral-bending range of motion, (B) cervical rotation range of motion, (C) glenohumeral internal rotation angle, (D) shoulder horizontal adduction angle.

Pain and nonpain lateral bending were measured with the iPhone on the contralateral head side with the level aligned with the eyes (Fig. 3). The starting position was sitting. The iPhone's level was aligned with the corner of the eye using the Clinometer.^[59]^ The subject was instructed to flex laterally as far as possible. For the cervical rotation angle, the pain and nonpain side rotation was measured with the iPhone placed on the participant's head with the arrow of the Compass application aligned with the nose (Fig. 3).^[59]^ After being stabilized, the subject had belts placed to prevent any trunk and shoulder movements during the performance of the cervical-lateral bending and rotation movement. For the frontal and transverse planes, measurements were made for the total range: the difference between the final and initial measures. The side difference was calculated by subtracting the nonpain side value from that of the pain side for the lateral-bending and rotation angles.

##### 2.2.5.2 Glenohumeral internal rotation angle

GIR ROM was measured in the supine position with the shoulder in 90° abduction and the elbow in 90° flexion (Fig. 3).^[29,60,61]^ Examiners maintained the subject's shoulder at 90° of abduction while measuring GIR ROM. The independent observer measured the GIR ROM data displayed by the Clinometer at the distal one-third of the subject's elbow when the examiners determined the endpoint of GIR ROM.

##### 2.2.5.3 Shoulder horizontal adduction angle

Shoulder horizontal adduction angle was measured in a supine position with the shoulder in 90° flexion and the elbow in 90° flexion (Fig. 3).^[62,63]^ With one hand, the clinician grasped the elbow of the tested side arm and passively abducted the humerus to 90° while maintaining 0° of rotation of the humerus and 90° of elbow flexion. The clinician ceased the movement when he felt that the humerus or scapula could no longer be stabilized or when movement stopped while passively moving the humerus into horizontal adduction.

#### 2.2.6 Strength measurements

##### 2.2.6.1 Serratus anterior strength

**Figure 4:**
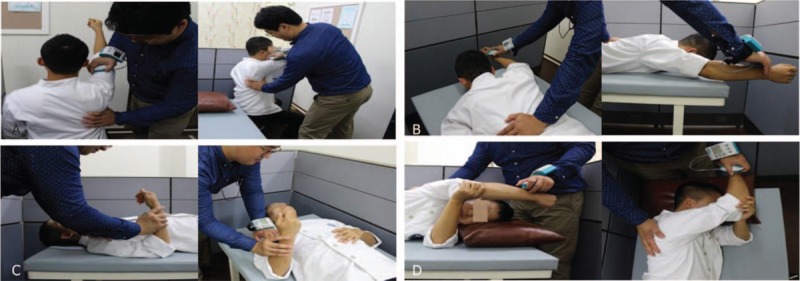
Measurement of muscle strength: (A) serratus anterior, (B) lower trapezius, (C) biceps, (D) glenohumeral external rotator.

For testing SA, participants were seated in a standard chair with their feet flat on the floor and back supported by the back rest. The arm was positioned with scapular protraction and the shoulder flexed to 125° (Fig. 4).^[64,65]^ Participants were asked to maintain the upper extremity position as the examiner provided a downward force with the dynamometer just over the distal humerus.

##### 2.2.6.2 Lower trapezius strength

The participant was given instructions regarding the test procedure and then placed in a prone position, with the upper extremity diagonally overhead, in line with the fibers of the LT (Fig. 4).^[66]^ To avoid compensation during the test, the examiner provided manual fixation by placing one hand just inferior to the subject's contralateral scapula and instructed the subject to maintain the cervical spine in a neutral position. The dynamometer force sensor was applied to the distal one-third of the subject's radial forearm, and force was applied by the examiner in a downward direction, toward the floor.

##### 2.2.6.3 Biceps strength

For stabilization, the examiner stood holding the subject's ipsilateral shoulder. The intra-rater and inter-rater reliability of testing was clearly increased by adding stabilization procedures to the supine position for bicep strength measurement (Fig. 4).^[67]^ The dynamometer force sensor was applied to the distal one-third of the subject's forearm, and force was applied by the examiner on the inferior side of subject until the subject's maximal muscular effort was overcome.

##### 2.2.6.4 Glenohumeral external rotator strength

To measure the glenohumeral external rotator strength, the subject lay on his or her side position with the shoulder flexed and internally rotated to 90° and the elbow flexed to 90° (Fig. 4). The subject supported the distal humerus of the measurement arm with the palm of the opposite hand. From the starting position, the subject moved to a position of full glenohumeral external rotation until the forearm was parallel with the table. Then, the dynamometer was applied to the distal one-third of the subject's radial forearm, and force was applied by the examiner in a downward direction, toward the floor, until the subject's maximal muscular effort was overcome.

### 2.3 Procedure

This study was performed for 9 months from March to November 2016. Subjects were evaluated at the work conditioning center in a theme park. The intra-rater reliability of measurements was examined by an orthopedic physical therapist with 4 years of clinical experience. The parameters were measured in the following order: psychological factors, posture, mobility, and strength. Subjects were instructed to complete a questionnaire (age, sex, VAS, BDI, and Borg BRPE scale) and then were photographed to measure posture. In order, 4 ROMs (cervical lateral-bending, rotation, GIR, and shoulder horizontal adduction) and 4 muscle strengths (SA, LT, biceps, and glenohumeral external rotator muscle) were measured.

### 2.4 Statistical analysis

The Kolmogorov–Smirnov Z test was used to assess the assumption of distribution normality. Descriptive statistics in all variables showed normal distributions. Pearson correlation matrices were constructed to examine the relationships between the VAS and the 16 variables. To investigate which psychological, postural, mobility, and strength variables contributed most significantly to the degree of UT pain, multiple regression models with a stepwise selection procedure were performed for the 16 independent variables, with VAS as the dependent variable. The determination coefficient (*R*^2^) showed the explanatory power for models of regression variables in multiple regression with a stepwise selection procedure.

Statistical analyses were conducted using SPSS (ver. 18.0) and the significance level was set at *P* = .05. We also performed post hoc power analyses using G∗power (ver. 3.1.2; Franz Faul, University of Kiel, Kiel, Germany) to confirm that the number of subjects was sufficient to achieve a large power. Effect sizes were chosen following the recommendations of Cohen.^[68]^

## 3 Result

All variables satisfied a normal distribution (*P* > .05). Table 2 shows the correlation coefficient between the VAS and age, sex (male = 0 and female = 1), BRPE scale, BDI, FHP angle, RSA, SSA, SDRR, cervical lateral-bending side difference angle, cervical rotation side difference angle, GIR angle, shoulder horizontal adduction angle, SA strength, LT strength, bicep strength, and glenohumeral external rotator strength. There were significant negative correlations between VAS and SSA (*r* = −0.134; *P* < .05), SA strength (*r* = −0.695; *P* < .001), LT strength (*r* = −0.571; *P* < .001), bicep strength (*r* = −0.578; *P* < .001), and glenohumeral external rotator strength (*r* = −0.392; *P* < .001). There were positive correlations between VAS and age (*r* = 0.496; *P* < .001), BRPE scale (*r* = 0.553; *P* < .001), FHP angle (*r* = 0.168; *P* < .05), RSA (*r* = 0.292; *P* < .001), SDRR (*r* = 0.200; *P* < .05), and GIR angle (*r* = 0.211; *P* < .05). No significant correlations were found between the VAS and sex, cervical lateral-bending side difference angle, the cervical rotation side difference angle, and shoulder horizontal adduction angle (*P* > .05).

**Table 2:**
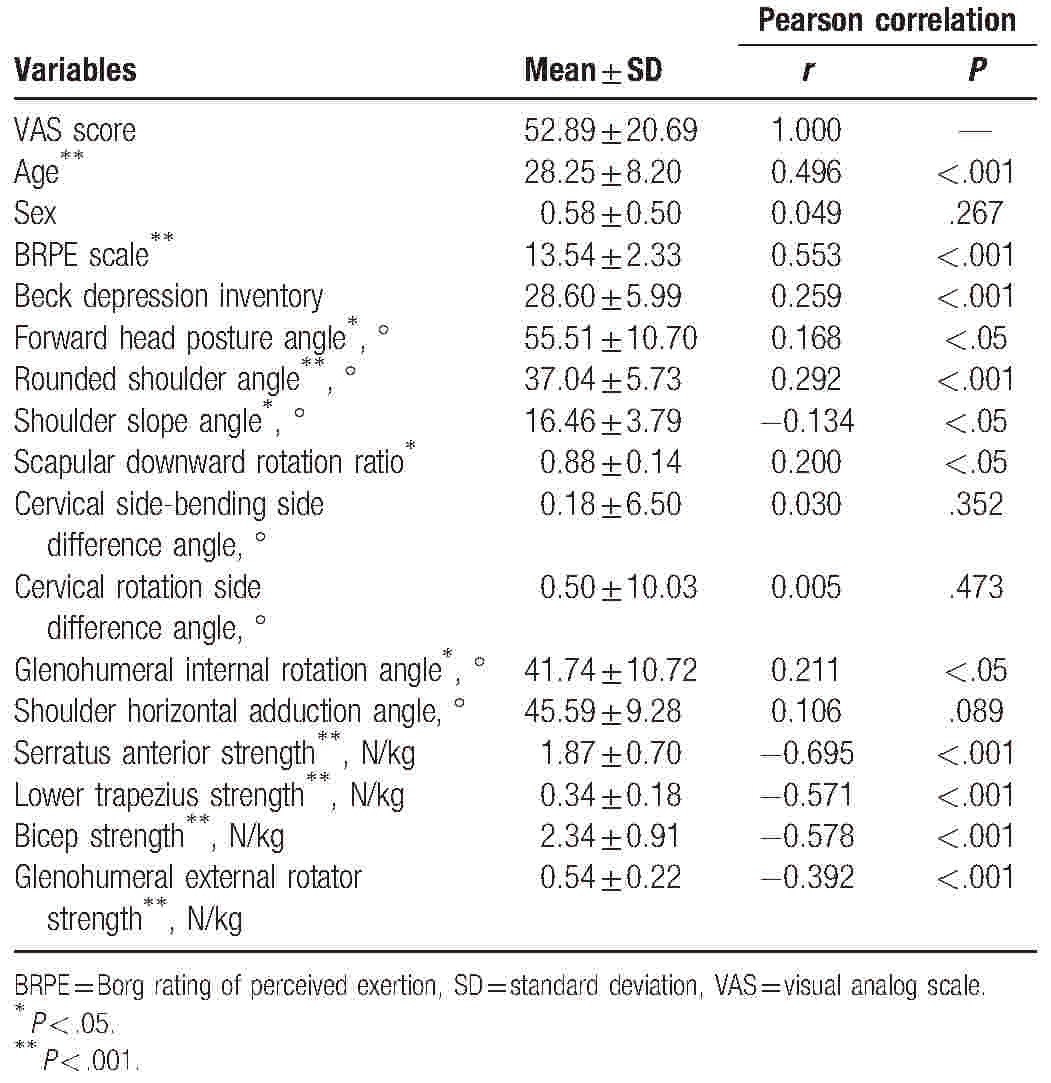
Descriptive statistics for variables and results of Pearson correlation.

Stepwise multiple-regression analyses were performed to identify variables that contributed significantly to VAS in FSWs with UT pain for MTrPs. In the stepwise regression analyses, model 5 included SA strength, age, BRPE scale, LT strength, and RSA as predictor variables and accounted for 68.7% of the variance in VAS (Table 3; *P* < .001).

**Table 3:**
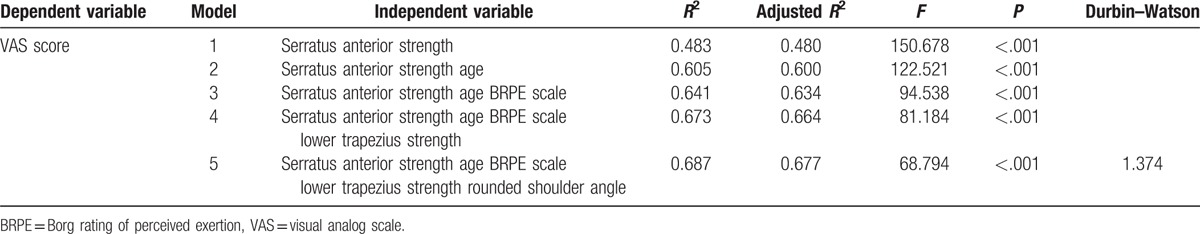
Results of stepwise multiple regression analyses for models.

Unstandardized and standardized coefficients are shown in Table 4. According to the independent variables, the regression equations were set up using slope and constant values in unstandardized coefficients. The VAS was computed using the regression equation. In β values, as standardized coefficients of model 5, the following were independent influencing variables on VAS, in order: SA strength (β = −0.380), age (β = 0.287), BRPE scale (β = 0.239), LT strength (β = −0.195), and RSA (β = 0.125).

**Table 4:**
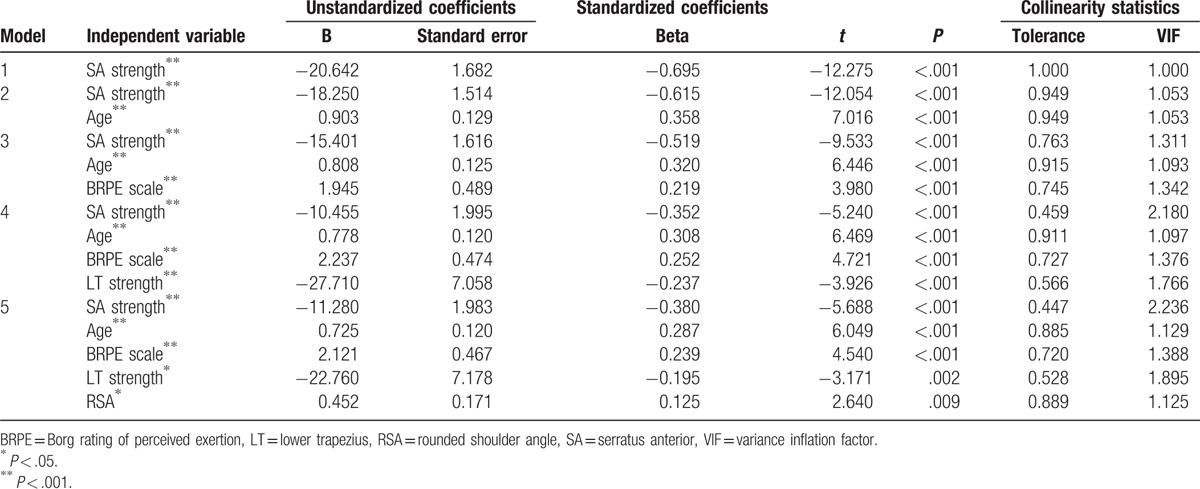
Results of stepwise multiple regression analyses for coefficients of independent variables in models.

Post hoc power analyses were calculated by setting the significance level *P* = .05, total sample size = 163, number of predictors = 17, and effect size f^2^ = 2.19 (by calculating from *R*^2^ = 0.687 in model 5). The power value was computed to be 1.00. Thus, the post hoc power analysis confirmed that the power was sufficient for multiple regression.

## 4 Discussion

We investigated psychological, posture, mobility, and strength factors associated with UT pain with MTrPs in FSWs. The examination of shoulder posture, mobility, and strength is important for a treatment approach to shoulder pain. However, although the population of FSWs has increased with the growth of the food service industry, to the best of our knowledge, there has been no previous report on which factors are related to shoulder pain. Here, we demonstrated that SA strength, age, BRPE scale, LT strength, and RSA were significant predictors of UT pain with MTrPs in FSWs. These results can help in the design of treatment or exercises to decrease UT pain with MTrPs in FSWs.

SA strength showed a significant correlation with VAS of UT pain, accounting for 48.3% of the variance (*P* < .001) in the resulting model 1. The SA contributes to scapular movements such as protraction, upward rotation, and posterior tilt.^[69]^ In addition, the SA performs the role of a scapular upward rotator and a scapular dynamic stabilizer during humeral elevation.^[23,40]^ Because FSWs frequently carry food or food materials and very often lift cooking equipment, humeral elevation occurs with high frequency. SA weakness^[33]^ and muscle imbalance in the scapulothoracic and glenohumeral joints^[22,31]^ can lead to shoulder dysfunction and an abnormal scapulohumeral rhythm or scapular dyskinesis.^[70]^ This is possibly the reason why SA weakness leads to overuse of the UT and causes UT pain. In particular, excess activation of UT has been proposed as contributing to abnormal scapular motion.^[23,40]^ In subjects with UT pain, overload on the UT for excessive activation compensated for a weakened SA muscle.^[71]^ By contrast, Lucas et al^[72]^ suggested that the MTrPs are associated with changes in motor control prior to the presence of pain. The cause and effect relationship between UT pain and SA strength has been controversial.^[22,31,40,72]^ Although this study found a clear association between UT pain and SA strength, the cross-sectional study design does not allow inferences about a possible causal relationship. Therefore, it is possible that pain influenced strength rather than the other way around. Because the B value of the unstandardized coefficient for SA strength was −20.642 in model 1, a regression equation with negative slope was set. A negative slope may mean that UT pain decreases according to increases in SA strength. Thus, SA strengthening may be a treatment for decreasing UT pain with MTrPs in FSWs.

In model 2 (*P* < .001), the combination of SA strength and age showed a significant correlation with the VAS of UT pain, accounting for 60.5% of the variance. The onset of MTrPs may be initiated by repetitive microtrauma, including the overuse and overloading of muscles, which often heighten chronic MTrPs.^[73]^ The aging degeneration of the musculoskeletal system, with the gradual loss of myofascial flexibility, is a source of vulnerability and eventually results in active MTrPs.^[74–76]^ Consequently, increased age may also be associated with an increased number of MTrPs.^[73–76]^ FSWs are exposed to repetitive manual work for long periods causing microtrauma and muscle fatigue, and perform forceful movements and lift weights in awkward working postures. As the B value of the unstandardized coefficient for age was 0.903 in model 2, a regression equation with a positive slope was set. A positive slope may mean that UT pain increases with increasing age for FSWs.

The current findings show that the addition of BRPE scale increased the predictive value of the VAS of UT pain by 3.6% in the resulting model 3 (*P* < .001). In a report by Dempsey and Filiaggi,^[77]^ the mean rating of the BRPE scale was 10.3 in 85 FSWs working at casual dining restaurants located in the eastern United States. This study showed that the perception of exertion for food service tasks was rated as “somewhat hard” (mean ± standard deviation [SD]: 13.54 ± 2.33) by FSWs working at restaurants in a theme park, and the ratings on the BRPE scale in this study were greater than those of the previous study.^[77]^ About 90% of visitors to theme parks require food service facilities.^[78]^ Furthermore, theme parks show a wide range of food service facilities for adults actively involved in the attractions as well as for those passively participating in their children's entertainment.^[79]^ Due to these characteristics of a much-frequented food restaurant and many different kinds of restaurants in the theme park, the values attained on the BRPE scale in the present study may be higher than those in previous studies. Because the B value of unstandardized coefficients for the BRPE scale was 1.945 in model 3, a regression equation with positive slope was set. This may indicate that UT pain could increase in accordance with increasing workload.

We also found that the combination of model 4, SA strength, age, BRPE scale, and LT strength resulted in a 3.2% greater predictive value in VAS of UT pain (*P* < .001). Scapulothoracic muscle imbalances result in impaired biomechanics and pain.^[22,36,37]^ Muscle imbalance is described as an impaired relationship between muscles prone to tightness that lose extensibility and those prone to inhibition and weakness.^[80]^ A previous study demonstrated a significant difference in LT strength between the ipsilateral (mean ± SD: 21.8 ± 10.0 N) and contralateral sides (mean ± SD: 25.7 ± 11.5 N) in pain in individuals with unilateral neck and shoulder pain.^[81]^ In the present study, LT strength was 0.34 ± 0.18 N/kg. Before LT strength was divided by body weight, LT strength was 21.56 N. Thus, the measured LT strength in the present study was similar to the results of a previous study in subjects with UT pain. The musculature biomechanically linked to an area of pain could potentially be weaker on the symptomatic side, or the process could work in the reverse way. Because the B value of unstandardized coefficients for LT strength was −27.710 in model 4, a regression equation with negative slope was set. The negative slope suggests that UT pain decreased in accordance with increased LT strength.

In the resulting model 5, the combination of SA strength, age, BRPE scale, LT strength, and RSA explained an additional 1.4% of the variance in the VAS of UT pain (*P* < .001). An abducted or forward scapula or a rounded shoulder lengthens the UT,^[23,82]^ resulting in a decreased PPT, in accordance with increasing tension in the UT.^[21]^ In the normal alignment of the scapula in the transverse plane, it is tilted 30° in the anterior to frontal plane.^[83]^ This study confirmed that RSA was 37.04° ± 5.73° (mean ± SD) in FSWs and the measured RSA was 7.04° greater than this normal alignment. There are 2 possible reasons why UT pain may cause an increase in RSA. First, the rhomboids and middle trapezius muscle could lengthen with an increasing RSA. The rhomboids and middle trapezius muscle are scapular stabilizers.^[9,23]^ Lengthening these muscles might make it difficult for them to perform at optimal muscle strength as scapular stabilizers. Second, the altered RSA could increase scapular medial rotation. Because the altered scapular alignment or position can decrease muscle strength^[84,85]^ and alter neuromuscular patterns and scapulohumeral rhythm,^[22]^ this might affect UT pain. As the B value of the unstandardized coefficients for RSA was 0.452 in model 5, a regression equation with a positive slope was set. Thus, this study demonstrated that UT pain may increase with an increasing RSA.

A “scale-free” standardized coefficient may be more meaningful than an unstandardized coefficient to compare independent variables. The standardized coefficients in order of absolute value are as follows: SA strength (β = −0.380), age (β = 0.287), BRPE scale (β = 0.239), LT strength (β = −0.195), and RSA (β = 0.125). This order could be interpreted as the order of influence on UT pain with MTrPs. In addition, the assessment of SA strength, age, BRPE scale, LT strength, and RSA could predict the amount of UT pain through a multiple regression equation: 
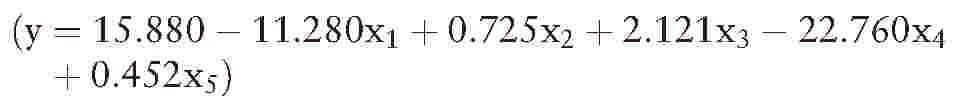


Several limitations of this study should be noted. First, the sample sizes were modest and there was little ethnic diversity, which limits the generalizability of the findings. Second, this study had a cross-sectional design. Therefore, further longitudinal study needs to confirm any causal relationship between the psychological, posture, mobility, and strength factors and pain severity.

## 5 Conclusions

The assessment of SA strength, BRPE scale, LT strength, RSA, and SSA would be able to predict the amount of UT pain through a multiple regression equation. In addition, the results of this investigation may be useful for developing guidelines for treatments or interventions for UT pain.

## Acknowledgment

This work was supported by the Yonsei University Research Fund of 2017-51-0018 and Brain Korea 21 PLUS Project (Grant NO. 2016-51-0009) sponsored by the Korean Research Foundation for Department of Physical Therapy in Graduate School, Yonsei University. The authors wish to express sincerely appreciation to all voluntary participants in the study.
